# Integrative Physiological and Transcriptomic Analysis Reveals Metabolic Adaptation and Cold-Tolerance Marker Development in Winter Rye Under Low-Temperature Stress

**DOI:** 10.3390/plants14111588

**Published:** 2025-05-23

**Authors:** Haonan Li, Jiahuan Zhao, Chenguang He, Yang Guan, Huimin Guan, Ting He, Dexu Meng, Xiaoping Wang, Yimiao Tang

**Affiliations:** 1 Key Laboratory of Molecular Cell Genetics and Genetic Breeding in Heilongjiang Province, College of Life Science and Technology, Harbin Normal University, Harbin 150025, China; ryan_lhn@163.com (H.L.); zhaojh001201@163.com (J.Z.); 1880045389@163.com (C.H.); guanguan0804@126.com (H.G.); ht15772790717@163.com (T.H.); 18645758939@163.com (D.M.); 2 Heilongjiang Provincial Laboratory of Plant Physiology, College of Life Science and Technology, Harbin Normal University, Harbin 150025, China; guany0611@126.com; 3 Beijing Key Laboratory of Crop Molecular Design and Intelligent Breeding, Beijing Key Laboratory of Molecular Genetics in Hybrid Wheat, Institute of Hybrid Wheat Beijing Academy of Agriculture and Forestry Sciences, Beijing 100097, China

**Keywords:** rye (*Secale cereale*), low-temperature stress, metabolism, molecular markers

## Abstract

Rye (*Secale cereale*), a cereal crop with high cold tolerance, serves as an ideal model for investigating plant cold adaptation mechanisms. Despite recent progress in identifying numerous genes and metabolic changes associated with cold tolerance, the detailed regulatory networks and coordinated interactions between metabolic pathways under low-temperature stress in rye remain unclear. In this study, we focused on the winter rye variety “Winter” and systematically explored its metabolic regulatory responses to cold stress through a combination of low-temperature treatments, phenotypic observations, antioxidant enzyme activity assays, and transcriptomic analysis. Four rye varieties (“Winter”, HZHM3, HZHM8, and “Victory”) were compared for cold tolerance, with the results indicating that “Winter” and HZHM3 exhibit superior cold tolerance. Physiological analysis revealed that after 12 h of exposure to −4 °C, the activities of catalase (CAT), peroxidase (POD), and ascorbate peroxidase (APX) in “Winter” were significantly upregulated, displaying an initial increase followed by a decline over time. Transcriptomic sequencing identified 1643 differentially expressed genes (DEGs), and GO, KEGG, and GSEA enrichment analyses highlighted the critical roles of carbohydrate metabolism (ko00630) and amino acid metabolism (ko00250) pathways in the cold stress response. These pathways are interconnected through key metabolic intermediates such as L-glutamate, collectively regulating cold adaptation. Furthermore, based on the transcriptomic data, we identified and developed molecular markers associated with cold tolerance, detecting 10,846 EST-SSR and 250,116 EST-SNP markers. We successfully developed 13 EST-SSR primer pairs applicable to rye and 7 KASP markers. Notably, the KASP-665 marker effectively distinguishes between winter and spring rye, providing a reliable tool for marker-assisted selection in cold tolerance breeding. This study not only elucidates the metabolic regulatory mechanisms of rye under low-temperature stress but also provides a solid theoretical and technical foundation for future cold-tolerance breeding programs.

## 1. Introduction

Low temperature is a critical abiotic stress factor that severely restricts plant development and crop yield [1,2]. Exposure to low temperatures reduces enzyme activities and slows metabolic processes, thereby disrupting cellular homeostasis and ultimately leading to yield loss. In response to cold stress, plants undergo a series of physiological adjustments, including modifications in membrane composition to optimize substance transport and energy metabolism, an increase in reactive oxygen species (ROS) levels that triggers oxidative stress, and alterations in antioxidant enzyme activities (such as SOD and POD) to mitigate ROS damage [3,4]. For instance, rice seedlings exposed to low temperatures of 10–12 °C show a rapid increase in SOD and POD activities during the initial phase of stress, while barley exhibits a marked upregulation of these enzymes under conditions ranging from −2 °C to 2 °C, although POD activity may decline as stress intensifies [5,6]. Similar adaptive mechanisms have been observed in maize and other crops.

In recent years, the advent of RNA-sequencing technology has enabled a comprehensive analysis of gene expression changes in plants under cold stress [7,8]. This approach not only captures protein-coding mRNAs but also includes various non-coding RNAs and splice variants, providing a robust tool for elucidating regulatory networks and molecular mechanisms underlying cold tolerance [9,10]. Moreover, high-throughput sequencing has significantly advanced the development of functional molecular markers—such as EST-SSR, EST-SNP, and KASP markers—which are crucial for assessing genetic diversity and facilitating marker-assisted breeding [11,12].

Rye (*Secale cereale*), known for its superior cold tolerance, serves as an ideal model for investigating plant cold adaptation mechanisms. Recent studies on cold tolerance in rye have yielded significant insights; for example, transcriptomic analyses under low-temperature stress have revealed critical roles for the CBF (C-repeat binding factor) transcription factor family and their downstream *COR (Cold-regulated)* genes, along with notable alterations in carbohydrate and amino acid metabolism [13,14]. However, the regulatory networks among these genes and the coordinated interactions between different metabolic pathways under cold stress remain incompletely understood.

In this study, we focus on the winter rye variety “Winter” and combine low-temperature treatments, phenotypic assessments, antioxidant enzyme activity assays, and transcriptomic analyses to systematically elucidate the metabolic regulatory mechanisms involved in cold response. Furthermore, we aim to develop molecular markers associated with cold tolerance. By comparing the cold tolerance of four rye varieties (“Winter”, HZHM3, HZHM8, and “Victory”), this work seeks to provide new insights into the comprehensive regulatory network of cold tolerance in rye and to support the advancement of marker-assisted breeding for cold-tolerant cereal crops.

## 2. Results

### 2.1. Physiological Responses and Cold Tolerance Evaluation of Different Rye Varieties Under Low-Temperature Stress

**Figure 1:**
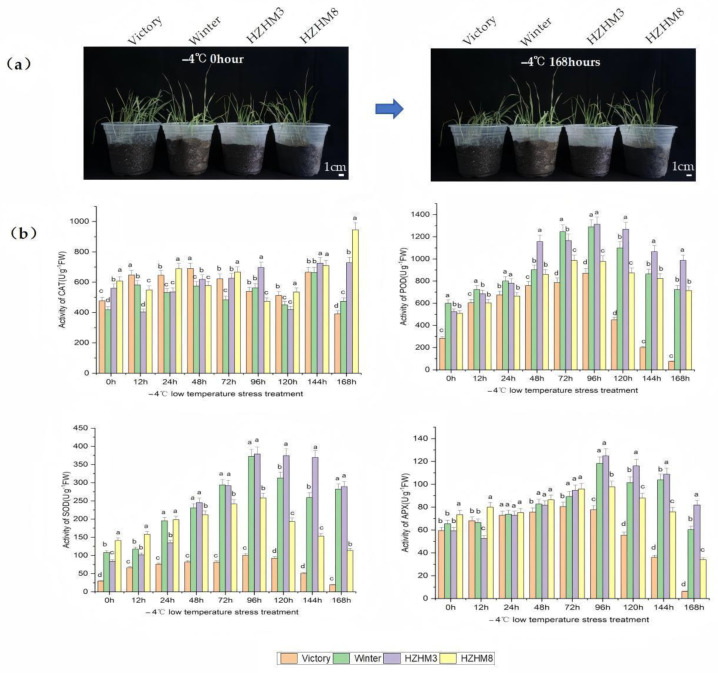
Phenotypic and physiological responses of four rye varieties to low-temperature stress. (**a**) Phenotypic changes in four rye varieties (“Winter”, HZHM3, HZHM8, and “Victory”) following low-temperature treatment. Growth status at 0 h and 168 h of treatment. (**b**) Histograms depicting physiological responses of the four rye varieties under low-temperature stress, including CAT, POD, SOD, and APX activity levels. Different letters indicate significant difference at *p* < 0.05. Error bars are the standard deviation.

To evaluate the cold tolerance of four rye varieties—“Winter”, “Victory”, HZHM3, and HZHM8—this study analyzed their phenotypic changes and physiological responses under low-temperature stress, focusing on the activity dynamics of four key antioxidant enzymes: POD, SOD, CAT, and APX (Figure 1). The results showed that, except for “Victory”, which exhibited persistent wilting after 24 h of cold stress, the other three varieties displayed no significant phenotypic changes throughout the stress period (Figure 1a). In “Winter”, POD and APX activities remained stable at 12 h, while SOD, POD, CAT, and APX activities increased initially, peaking at 96 h before declining. The enzyme activity trends in HZHM3 were similar to those in “Winter” but slightly lower. Comprehensive analysis indicated that “Winter” and HZHM3 exhibited strong cold tolerance (Figure 1b). Overall, the three winter rye varieties (“Winter”, HZHM3, and HZHM8) demonstrated significantly higher cold tolerance than the spring rye variety “Victory”, particularly in terms of SOD activity. These findings provide physiological insights into rye cold tolerance and valuable references for breeding cold-resistant rye varieties.

### 2.2. Transcriptome Analysis and Differentially Expressed Gene Screening in “Winter” Rye Under −4 °C Low-Temperature Stress

**Figure 2:**
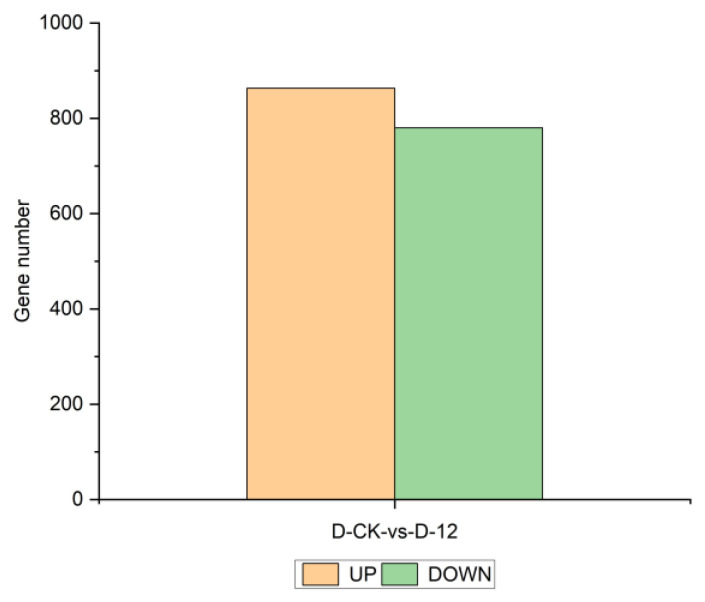
Statistical distribution of differentially expressed genes in the “Winter” rye transcriptome. Yellow bars indicate the number of upregulated genes in “Winter” rye under 12 h of cold stress compared to the control group, while green bars represent the number of downregulated genes under the same conditions.

Phenotypic and physiological analyses indicated that “Winter” rye exhibits strong cold tolerance under −4 °C low-temperature stress. To further investigate its cold tolerance mechanism, we conducted transcriptome sequencing to analyze gene expression profiles and relationships. Based on differential expression analysis, differentially expressed genes (DEGs) were identified, and their expression levels were examined. The filtered sequencing data for six samples are summarized in Table A1, showing that the quality of all clean reads exceeded 99.70%, indicating high data reliability. A total of 1643 DEGs were identified, including 863 upregulated and 780 downregulated genes (Figure 2).

### 2.3. GO and KEGG Enrichment Analysis of Differentially Expressed Genes in “Winter” Rye Under Low-Temperature Stress

**Figure 3:**
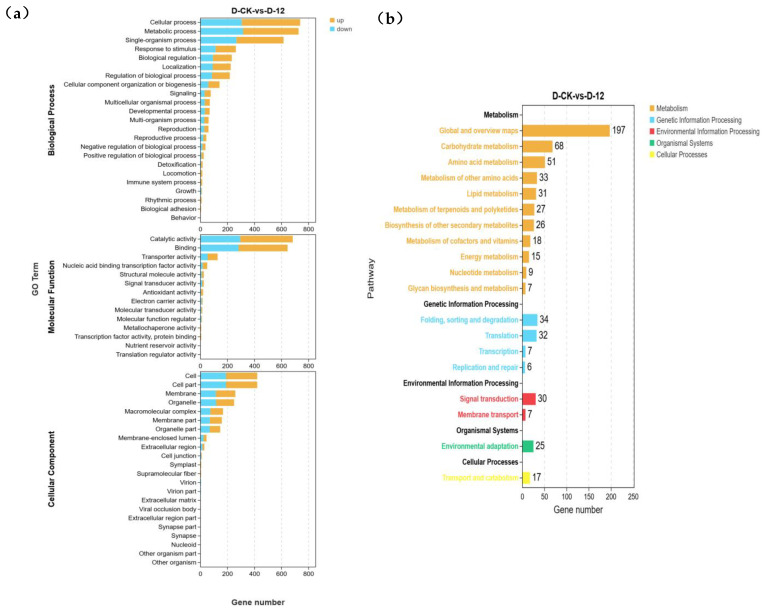

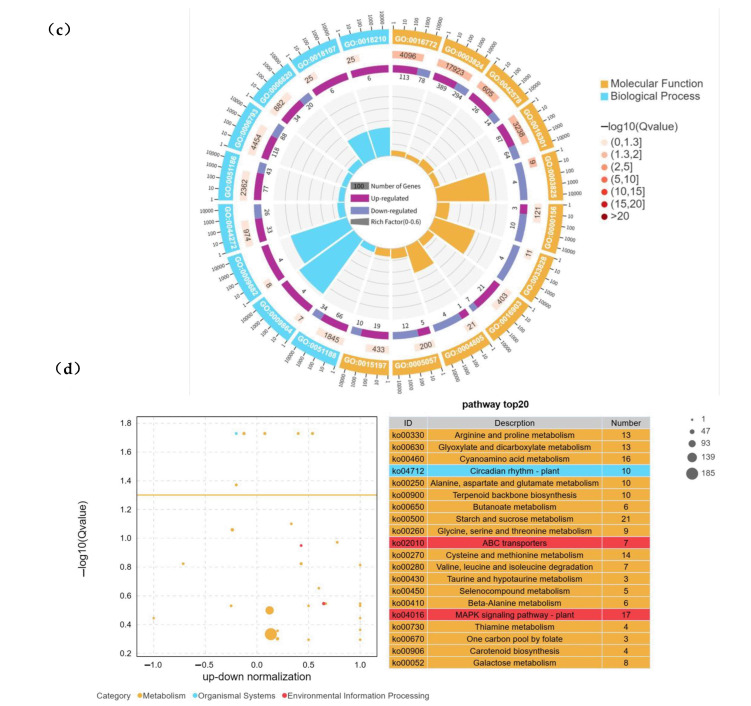
Transcriptomic differential gene expression changes in “Winter” rye under cold stress and a KEGG enrichment map of DEGs expression. (**a**) GO analysis of DEGs in “Winter” rye after 12 h of cold stress. The red bars represent upregulated DEGs, and the green bars represent downregulated DEGs. The vertical axis indicates the number of DEGs, while the horizontal axis corresponds to different biological functions, including biological processes, cellular components, and molecular functions. (**b**) KEGG pathway enrichment analysis of differentially expressed genes in the “Winter” rye transcriptome under cold stress. The diagram illustrates DEG enrichment across various pathways, including cellular pathways, genetic information, metabolism, and organic systems. The vertical axis represents the pathway names, and the horizontal axis shows the number of genes involved in each pathway. The length of each horizontal line corresponds to the number of DEGs in the pathway, with the color of the dots indicating different pathway categories. (**c**) Top 20 GO entry enrichments for differentially expressed genes in the transcriptome of “Winter” rye. This circular diagram displays the names or descriptions of GO entries in the outer circle, involving biological processes and molecular functions. In the middle circle, the distribution of upregulated and downregulated genes in each GO term is indicated. Purple domains represent upregulated gene enrichment, and blue domains represent downregulated gene enrichment. (**d**) Top 20 KEGG pathways enriched for differentially expressed genes in the “Winter” rye transcriptome. The vertical axis represents the pathway names, and the horizontal axis represents the number of differing genes. The size of the circles indicates the degree of DEG enrichment in each pathway, with larger circles representing more significant enrichment.

To further explore the functional roles of differentially expressed genes (DEGs) in “Winter” rye under low-temperature stress, we conducted Gene Ontology (GO) enrichment analysis (Figure 3a,b) and Kyoto Encyclopedia of Genes and Genomes (KEGG) enrichment analysis (Figure 3c,d). Figure 3a,b shows the overall GO annotations for the differentially expressed genes, covering a wide range of biological processes, cellular components, and molecular functions. In contrast, Figure 3c presents the top 20 GO terms based on the enrichment analysis results. These top 20 terms are selected from the broader set of GO annotations and are the ones that are most significantly enriched among the differentially expressed genes, providing a more focused view of the key biological functions affected by cold stress.

In the GO enrichment analysis, the most enriched biological process (BP) term was cellular process (GO:0009987), which included 432 upregulated and 306 downregulated DEGs, followed by metabolic process (GO:0008152). In the cellular component (CC) category, both cell (GO:0005623) and cell part (GO:0044464) exhibited an identical number of DEGs (420), indicating that these genes play roles in both cellular structures. In the KEGG enrichment analysis, 25 DEGs were identified, and they were primarily classified into two major pathways: circadian rhythm-plant (ko04712), with *PRR* genes predominantly enriched in this pathway, and plant–pathogen interaction (ko04626), with *RBOHF* genes being the main contributors.

Further examination of the top 20 KEGG pathways revealed that most of the DEGs were associated with metabolism, with a smaller subset enriched in environmental information processing pathways. Specifically, within metabolism, the pathways arginine and proline metabolism (ko00330), glyoxylate and dicarboxylate metabolism (ko00630), cyanoamino acid metabolism (ko00460), and alanine, aspartate, and glutamate metabolism (ko00250) showed the highest levels of enrichment. In the environmental information processing category, significant enrichment was observed in ABC transporters (ko02010) and the MAPK signaling pathway—plant (ko04016). These results provide important insights into the molecular mechanisms underlying “Winter” rye’s response to low-temperature stress and offer a foundation for further investigation into cold tolerance mechanisms.

### 2.4. KEGG and GSEA Analysis of Differentially Expressed Genes (DEGs) in “Winter” Rye Under Cold Stress and Identification of Key Metabolic Pathways

**Figure A1:**
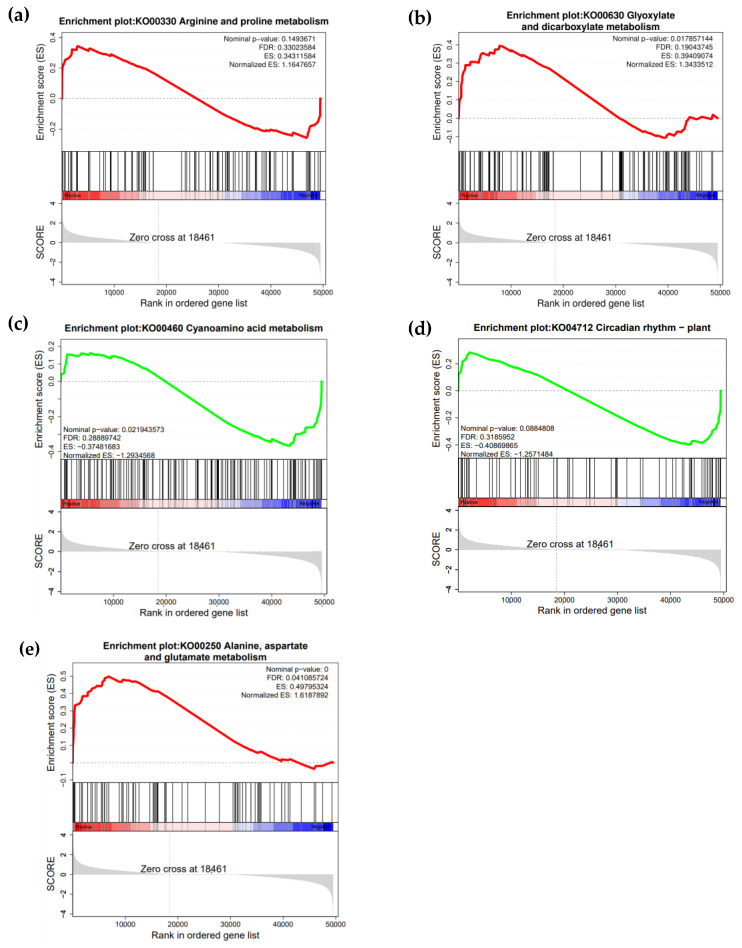
GESA plots of genes for the top 5 items with the highest KEGG enrichment (q-value = 0.018685). (**a**) GESA plot of KO00330; (**b**) GESA plot of KO00630; (**c**) GESA plot of KO00460; (**d**) KO04712 GESA plot; (**e**) KO00250 GESA plot. The upper part of the figure represents the distribution curve of the enrichment pathway name and the ES value of each position, the vertical axis represents the enrichment score, the upper right corner NES represents the corrected normalized ES, P.adj represents the significance of the gene set enrichment, the FDR represents the *p* value corrected by the FDR method, the middle part represents the position of genes in the gene set S in the gene sorting list L, and each vertical line represents a gene in the pathway. The lower part represents the normalized values of genes in the gene sorting list L for visualization. Note: It is generally considered that |The NES| > 1, Padj < 0.05, FDR < 0.25 pathways were significantly enriched.

To identify significant gene sets associated with differentially expressed genes (DEGs), we conducted gene set enrichment analysis (GSEA) on the top five most enriched KEGG pathways (q-value = 0.018685). Interestingly, the pathways glyoxylate and dicarboxylate metabolism (ko00630) and alanine, aspartate, and glutamate metabolism (ko00250) met the threshold for significant gene set enrichment. Both pathways are involved in carbohydrate metabolism and amino acid metabolism within the broader metabolic category (Figure A1).

**Figure 4:**
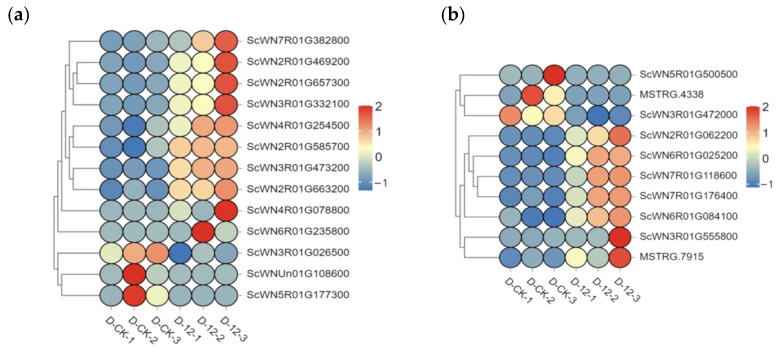
Heat map of differential gene expression in the glyoxylate and dicarboxylate metabolism and alanine, aspartate, and glutamate metabolism pathways under cold stress. (**a**) Changes in the expression of differentially expressed genes (DEGs) in the glyoxylate and dicarboxylate metabolism pathway under cold stress at 0 h and 12 h. (**b**) Changes in the expression levels of DEGs in the alanine, aspartate, and glutamate metabolism pathway under cold stress at 0 h and 12 h. Red indicates a significant upregulation of gene expression, while blue indicates significant downregulation. The horizontal axis represents the duration of cold stress treatment (0 h and 12 h), and the vertical axis represents the names of the differential genes.

We analyzed the genes responsible for the glyoxylate and dicarboxylate metabolism pathway, which contains 106 annotated genes. Of these, 13 genes were identified as candidates, including the *upregulated Serine hydroxymethyltransferase (SHM)* gene family, *Acyl-activating enzyme (AAE)* gene, and *downregulated Phosphoglycolate Phosphatase (PGLP)* gene and *Acetyl-CoA acetyltransferase (AACT)* gene (Figure 4a and Table A2).

In comparison to the glyoxylate and dicarboxylate metabolism pathway, the enrichment of the alanine, aspartate, and glutamate metabolism pathway was even more pronounced. Among the 10 candidate genes in this pathway, 7 were upregulated, and 3 were downregulated. Notably, the *Glutamate dehydrogenase (GDH)* gene (*ScWN5R01G500500*), *MSTRG.4338*, and *Amidophosphoribosyltransferase (ASE)* gene *(ScWN3R01G472000)* were significantly downregulated, while the other genes were upregulated. Importantly, compared to the control group, the *L-aspartate oxidase (NadB)* gene (*ScWN2R01G062200*) showed a remarkable increase in expression, upregulated approximately 10-fold under cold stress, while the *ASE1* gene (*ScWN3R01G472000*) was downregulated by about 3-fold (Figure 4b and Table A2).

**Figure 5:**
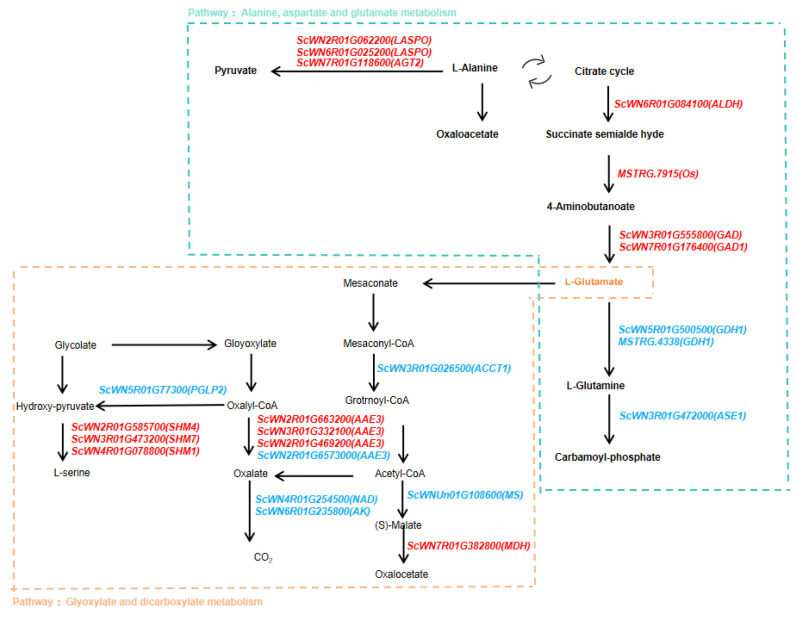
Pathway interactions between glyoxylate and dicarboxylate metabolism and alanine, aspartate, and glutamate metabolism under cold stress. The glyoxylate and dicarboxylate metabolism pathway is represented in orange, while the alanine, aspartate, and glutamate metabolism pathway is shown in blue-green. Genes highlighted in red indicate upregulated expression, whereas genes marked in blue indicate downregulated expression within their respective pathways.

To further elucidate the genetic regulatory mechanisms underlying “Winter” rye’s response to cold stress through the glyoxylate and dicarboxylate metabolism and alanine, aspartate, and glutamate metabolism pathways, we propose a working model for the interaction of these two pathways in response to cold stress (Figure 5). These pathways primarily interact through the synthesis and interconversion of intermediate metabolites such as L-glutamine, L-glutamate, and mesaconate, forming a complex network of carbohydrate and amino acid metabolism. Specifically, L-glutamate serves as a central product linking the two cascades, contributing to the downstream synthesis of oxalate and oxaloacetate in carbohydrate metabolism, while simultaneously supporting the synthesis of carbamoyl-phosphate in amino acid metabolism. Together, these two pathways play a coordinated role in “Winter” rye’s response to cold stress.

### 2.5. qRT-PCR Validation of Cold-Responsive Gene Expression and Expression Trends in “Winter” Rye

**Figure 6:**
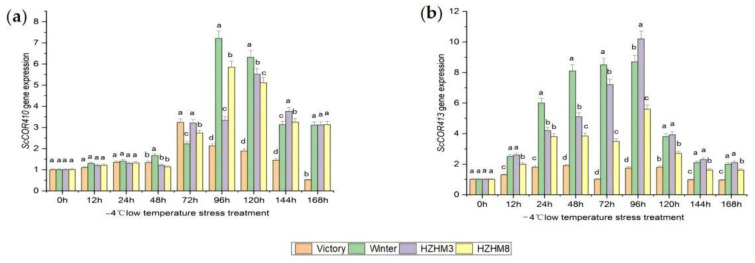
Expression dynamics of *ScCOR410* and *ScCOR413* in rye under cold stress. (**a**) Histogram illustrating the changes in *ScCOR410* expression levels in four rye varieties subjected to low-temperature stress. (**b**) Expression patterns of *ScCOR413* in four rye varieties under cold stress. Rye plants were subjected to 4 °C cold stress with sampling at 0 h, 12 h, 24 h, 48 h, 72 h, 96 h, 120 h, 144 h, and 168 h. Red bars indicate upregulated expression, while blue bars indicate downregulated expression. Different letters indicate significant difference at *p* < 0.05. Error bars are the standard deviation.

To validate the reliability of the transcriptomic data, we selected two *cold-regulation (COR)* genes and analyzed their expression trends under different durations of cold stress. The results showed that in “Winter” rye, *ScCOR413* expression increased approximately twofold after 12 h of cold stress and was significantly upregulated at 72 h and 96 h, reaching a peak expression level of nearly tenfold. Overall, the expression of *ScCOR413* exhibited an initial increase followed by a decline as the duration of cold stress prolonged (Figure 6).

Compared to HZHM8 and HZHM3, *ScCOR413* expression levels in HZHM3 were slightly higher at each time point, whereas at 96 h of cold stress, *ScCOR410* expression was higher in HZHM8 than in HZHM3; at other time points, HZHM3 maintained higher expression than HZHM8. Notably, “Victory” rye showed no significant expression of either gene at any time point, which contrasted with the expression patterns observed in the three winter rye varieties. This suggests that *ScCOR413* and *ScCOR410* are highly expressed exclusively in winter rye and may serve as candidate genes for cold tolerance.

### 2.6. Transcriptome-Based Development and Characterization of EST-SSR and KASP Markers for Cold Tolerance in Rye

**Figure A2:**
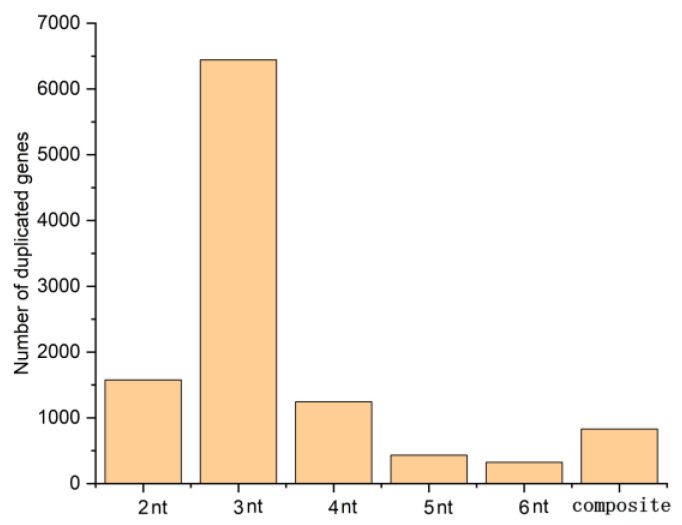
The number distribution of EST-SSR repeat unit types in “Winter”. The horizontal axis represents the type of repetitive unit, which is subdivided into repetitive units (2, 3, 4, 5, and 6) and composite units. The vertical axis corresponds to the number of each type of repeating unit.

**Figure A3:**
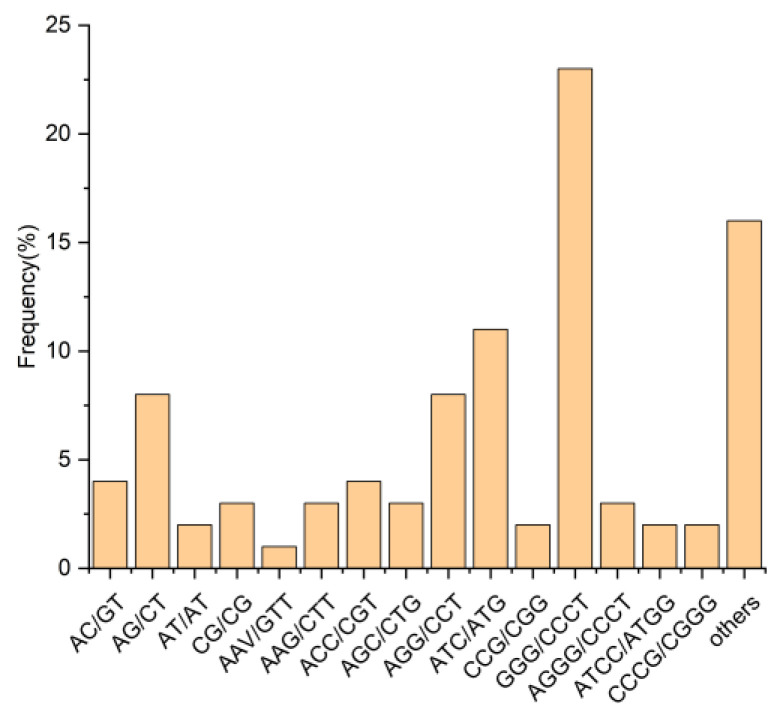
Distribution map of EST-SSR genotypes in “Winter”. The horizontal axis in the figure indicates different genotype markers, such as AC/GT, AT/AT, etc.; the vertical axis represents the relative number of these genotypes in the sample. The blue bars visually show the differences between genotypes.

Using transcriptomic data derived from the “Winter” rye cultivar, we identified a total of 10,846 EST-SSR loci (Table 1). Notably, chromosome 5R harbored the highest number of loci (1757), followed by chromosome 2R. Analysis of SSR repeat types revealed that dinucleotide, trinucleotide, and tetranucleotide repeats were predominant, accounting for 6442, 1576, and 1244 loci, respectively, while pentanucleotide, hexanucleotide, and compound SSRs were relatively infrequent (Figure A2). Among base repeat motifs, the CCG/CGG motif was most common (25.65%), followed by AGG/CCT (11.37%), AG/CT (8.32%), and AGGG/CCCT (1.52%) (Figure A3).

To assess the amplification efficiency of these EST-SSR markers, we randomly selected 151 primer pairs for testing across 31 rye accessions. Of these, 143 primer pairs successfully amplified target fragments, with 50 producing single, clear bands and 93 exhibiting polymorphism (Table A3). Importantly, 13 EST-SSR markers were universally amplified across all accessions, underscoring their potential as generic markers for rye (Table A4).

**Figure A4:**
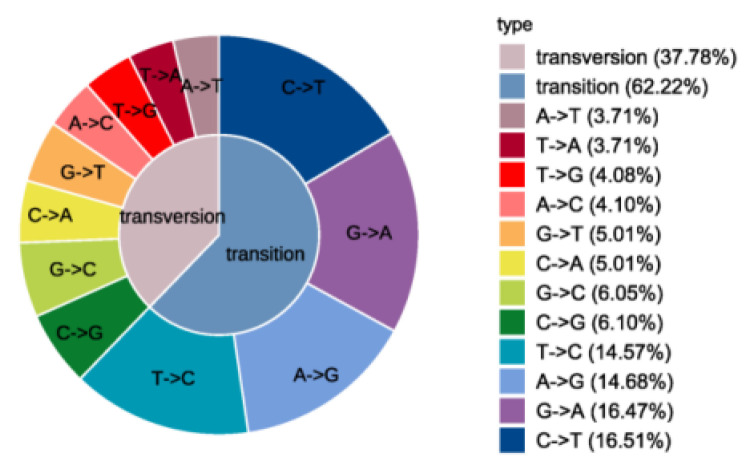
Statistics of “Winter” ESP-SNP mutants. Note: Base transversion and base transition; base transversion refers to the substitution between purine and pyrimidine in base substitution (G<->T, C<->A, A< ->T, C<->G, while base conversion is a purine replaced by another purine or a pyrimidine replaced by another pyrimidine (T<->C, A<->G).

Parallel SNP analysis of the transcriptomic dataset identified 250,116 SNPs. Base transition mutations accounted for 62.22% of these SNPs, while transversions comprised 37.78%. The most frequent SNP was a C→T transition (16.21%), followed by G→A, A→G, and T→C transitions, each exceeding 10% (Figure A4).

For Kompetitive Allele-Specific PCR (KASP) marker development, SNP-based screening was combined with qPCR validation. Three SNP loci, together with two *ScCOR4* genes and their corresponding SNP sites, were randomly selected. qPCR analysis revealed that *ScCOR4* genes were minimally expressed in spring rye under cold stress, indicating that these SNPs could serve as key molecular markers to distinguish between spring and winter rye accessions.

**Figure A5:**
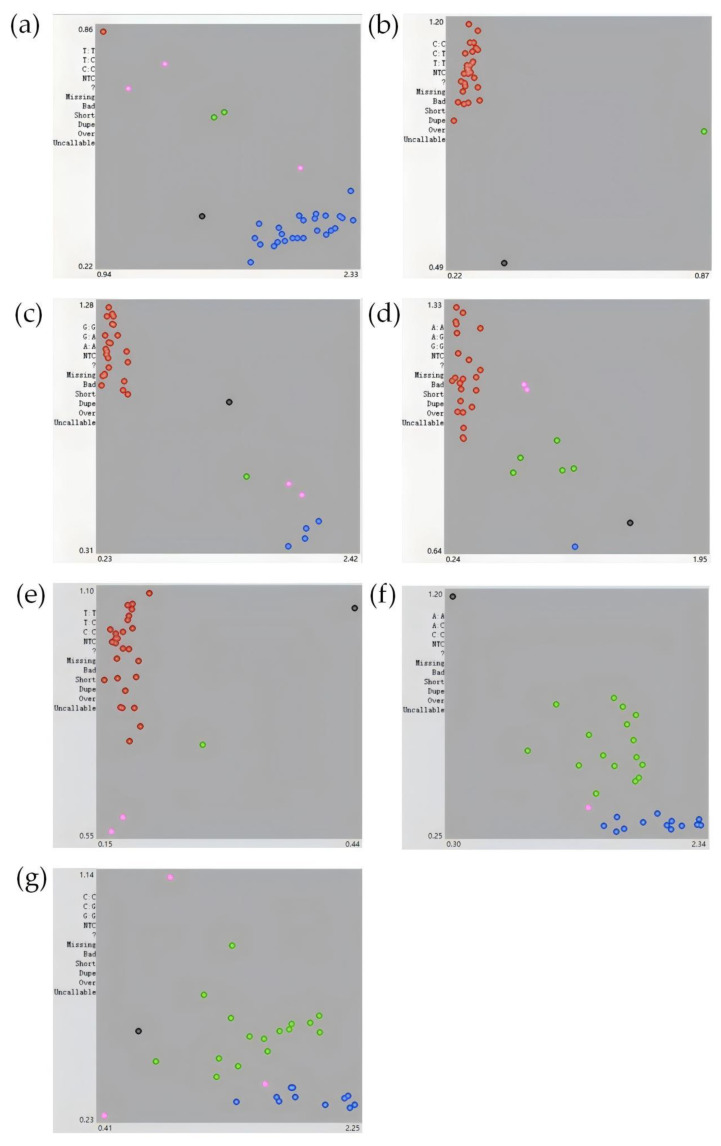
KASP marker genotyping results. (**a**) KASP-665—red dots are genotype T:T, green dots are genotype T:C, and blue dots are genotype C:C. (**b**) KASP-126—red dots are genotype C:C, green dots are genotype C:T, blue dots are genotype T:T, and black dots are NTC blank controls. (**c**) KASP-424—red dots are genotype G:G, green dots are genotype G:A, blue dots are genotype A:A, pink dots are untyped results, and black dots are NTC blank controls. (**d**) KASP-396—blue G:G, “Winter” rye, HZHM3, HZHM8, “Victory”, and RUS960; green A:G; pink for typing failure lines; red, genotype showing A:A; and black dots for NTC blank control. (**e**) KASP-063—the red dots are genotype T:T; green dots are genotype T:C; black dots are NTC blank control, and pink dots are untyped results. Each point within the axis represents a single plant individual. (**f**) KASP-050—blue dots are genotype C:C, green dots are genotype A:C, black dots are NTC blank control, and pink dots are untyped results. Each point within the axis represents a single plant individual. (**g**) KASP-109—blue dots are genotype G:G; green dots are genotype C:G, black dots are NTC blank control, and pink dots are untyped results. Each point within the axis represents a single plant individual.

Among the developed KASP markers, KASP-665 effectively differentiated the cold-tolerant cultivars “Winter” and HZHM8 (green signal, T:C) from the cold-sensitive cultivar “Victory” (red signal, T:T), with accessions of intermediate cold tolerance exhibiting a blue signal (C:C). As this SNP represents a transition mutation, KASP-665 serves as a specific and reliable marker for cold tolerance classification within this population (Figure A5).

Additionally, KASP-396 displayed distinct genotype signals: the blue signal (G:G) was exclusive to the RUS1090 cultivar; the green signal (A:G) was detected in “Winter”, HZHM3, HZHM8, “Victory”, and RUS960; and the red signal (A:A) was observed in the remaining 23 rye accessions. These genotype variations enabled clear classification of the 31 accessions into three distinct groups (Table A8). Furthermore, five additional KASP markers (KASP-424, KASP-396, KASP-063, KASP-050, and KASP-109) were developed for genetic population genotyping (Figure A5).

## 3. Materials and Methods

### 3.1. Plant Materials, Low-Temperature Stress Treatment, and Physiological Index Measurement

In this study, four rye varieties—“Winter”, “Victory”, HZHM3, and HZHM8—were selected. Uniform and plump seeds were germinated and grown in nutrient pots until the two-leaf stage under a controlled environment with a temperature of −4 °C, a relative humidity of 75%, and a 16 h light/8 h dark cycle. The seedlings were then subjected to low-temperature stress at −4 °C for different time intervals: 0 h, 12 h, 24 h, 48 h, 72 h, 96 h, 120 h, 144 h, and 168 h. After treatment, leaf samples (0.1 g) were collected, immediately flash-frozen in liquid nitrogen, and stored at −80 °C for further analysis. Leaf samples were collected from three separate plants for each treatment time point and variety, serving as three biological replicates to ensure the reliability and representativeness of the data.

Superoxide dismutase (SOD) activity was measured using the nitroblue tetrazolium (NBT) colorimetric method [15], peroxidase (POD) activity was determined using the guaiacol colorimetric method, catalase (CAT) activity was assessed using a visible light colorimetric method, and ascorbate peroxidase (APX) activity was measured using the ultraviolet absorption method for ascorbic acid activity determination [16]. All physiological indices were measured using a UV spectrophotometer. One-way analysis of variance (ANOVA) was performed using SPSS (version 25.0) software, and data visualization was carried out using Excel.

### 3.2. Transcriptome Sequencing

For transcriptome sequencing, “Winter” rye plants with uniform growth status were selected. Seedlings were subjected to −4 °C for 12 h (experimental group), while the control group was grown under normal conditions (a temperature of 22 °C, a relative humidity of 75%, and a 16 h light/8 h dark cycle. The 12-h timepoint was chosen for transcriptome sequencing because pilot experiments indicated that by this time, significant physiological changes had occurred in the rye seedlings in response to cold stress. These changes included notable alterations in antioxidant enzyme activities and gene expression patterns related to cold stress response pathways. Selecting this timepoint allowed us to capture the early-to-mid-stage molecular responses to cold stress, which are crucial for understanding the initial regulatory mechanisms activated in rye upon exposure to low-temperature stress. Moreover, it provided a balance between observing the immediate effects of cold stress and avoiding potential confounding factors that might arise from longer-term stress. Young leaves from both groups were collected, and total RNA was extracted using the Trizol reagent. mRNA was used as a template to synthesize double-stranded cDNA. The sequencing library was constructed through PCR amplification and enrichment of ligated products followed by paired-end sequencing (150 bp read length) on the Illumina HiSeq platform.

Raw sequencing reads were processed using Trimmomatic software (version 0.33) to remove adapter sequences and low-quality reads (reads with a base quality score < 20 and an N base proportion > 5%). Clean reads were aligned to the wheat reference genome using Hisat2 software (version 2.2.1), and StringTie software (version 2.2.3) was used for transcript reconstruction, novel transcript prediction, and the calculation of FPKM values to quantify gene expression levels.

### 3.3. Differentially Expressed Gene (DEG) Analysis and Functional Enrichment

Based on RNA-Seq data, gene expression levels were analyzed between the experimental and control groups. The input data consisted of a gene expression matrix (read counts), which was normalized using the EdgeR and DESeq2 software (version 1.38.3) packages. *p*-values and false discovery rates (FDR) were calculated, and genes with FDR < 0.05 and |log_2_FC| > 1 were considered significantly differentially expressed genes (DEGs).

Functional enrichment analysis of DEGs was performed using Gene Ontology (GO) and Kyoto Encyclopedia of Genes and Genomes (KEGG) pathway analysis to explore biological functions, molecular activities, and metabolic pathways. Significantly enriched pathways were visualized using bar plots based on *p*-values or Q-values.

### 3.4. Gene Set Enrichment Analysis (GSEA)

Gene set enrichment analysis (GSEA) was conducted using the GSEA-P software (version 4.2.3) and the MSigDB database to identify KEGG pathways significantly enriched between the experimental and control groups. The gene expression matrix was used as input, and gene ranking was performed using the signal-to-noise normalization method. Enrichment scores (NES) and *p*-values were calculated under default parameters. Significantly enriched pathways were determined based on the criteria |NES| > 1, P adj < 0.05, and FDR < 0.25.

### 3.5. qRT-PCR Validation

Quantitative real-time PCR (qRT-PCR) was used to analyze the expression of target genes using cDNA from “Winter”, “Victory”, HZHM3, and HZHM8 rye varieties at different low-temperature treatment time points (0 h, 12 h, 24 h, 48 h, 72 h, 96 h, 120 h, 144 h, and 168 h). The wheat actin gene *(TaActin)* was used as an internal reference (primer information is provided in Table A7). qRT-PCR reactions were conducted in two steps: Denaturation at 95 °C for 10 min. Amplification at 95 °C for 30 s, 60 °C for 30 s, 72 °C for 40 s, repeated for 39 cycles. The specificity of the amplification products was confirmed through melting curve analysis. Each experiment was performed in triplicate, and the 2^(−∆∆CT) method was used to calculate the relative expression levels of cold-responsive genes *ScWN5R01G457800* and *ScWN6R01G204000*.

### 3.6. Development of Rye EST-SSR Molecular Markers

MISA software (version MISA.pl, http://pgrc.ipk-gatersleben.de/misa/, accessed on 13 March 2022) was used to predict the EST-SSR loci of the already sequenced transcripts. The locus screening parameters were as follows: When 2, 3, 4, 5, and 6 bases were repeated for 6, 5, 4, 4, and 4 units, respectively, it was regarded as an SSR locus. If the distance between two SSRs sequences was less than 100 bp, it was considered a compound SSR. EST-SSR primers were designed using Primer3 software (version 1.1.4) with the following parameters: amplification product length, 100–300 bp; primer length, 18–25 nt; annealing temperature, 50–65 °C; GC content, 45–65%. A total of 151 primer pairs were selected and synthesized by Sangon Biotech. Using “Winter” rye transcriptome sequences, EST-SSR markers applicable to 31 rye varieties were developed.

### 3.7. Development of Rye EST-SNP and KASP Molecular Markers

EST-SNP loci in the transcriptome were identified using the Genome Analysis Toolkit (GATK) software(version 4.4.0.0). Based on SNP screening and qPCR validation, KASP molecular markers were developed. Three randomly selected SNP loci, along with two SNP loci from *ScCOR410* and two from *ScCOR413*, were used for KASP marker development. KASP reaction system (10 μL): 5 μL 2× KASP Master Mix; 0.14 μL Primer Mix; 4.86 μL Template DNA. KASP reaction conditions: Pre-denaturation at 94 °C for 15 min; denaturation at 94 °C for 20 s, annealing at 61–55 °C for 60 s, for 10 cycles; denaturation at 94 °C for 20 s, annealing at 55 °C for 60 s, for 26 cycles. Amplification was performed on a Bio-Rad CFX96 real-time PCR system, and the specificity of the products was confirmed through melting curve analysis.

## 4. Discussion

### 4.1. Antioxidant Enzymes and Cold Tolerance in Plants

Low-temperature stress induces a series of physiological and biochemical responses in plants, among which the modulation of the antioxidant enzyme system is crucial for mitigating cellular damage. Numerous studies have demonstrated that enhanced activities of antioxidant enzymes such as SOD, POD, and APX are closely correlated with improved cold tolerance [4,17,18,19,20,21,22,23,24,25]. In our study, phenotypic evaluations and measurements of antioxidant enzyme activities in different rye cultivars under cold stress revealed that the cold-tolerant cultivars “Winter” and HZHM3 exhibited the highest SOD, POD, and APX activities after 168 h of exposure. In contrast, the cold-sensitive cultivar “Victory” did not show a significant accumulation of these enzymes and displayed severe wilting. These findings underscore the importance of sustained and elevated antioxidant enzyme activities in conferring cold tolerance in rye.

Similar responses have been reported in other species. For instance, grapevine leaves exposed to 4 °C showed a transient increase in SOD, POD, and CAT activities, reaching a peak after 5–7 days of treatment [17,18,19]. Likewise, cold-tolerant rice cultivars rapidly increased the activities of SOD, POD, CAT, and APX under 5 °C stress, effectively scavenging reactive oxygen species and reducing lipid peroxidation, whereas cold-sensitive cultivars exhibited a slower and lower increase in enzyme activities. Comparable trends were observed in chickpea, where cold-tolerant genotypes maintained higher antioxidant activities during cold acclimation and freezing treatments compared to sensitive ones. Our study not only confirms the dynamic changes of antioxidant enzymes under cold stress in rye but also highlights “Winter” and HZHM3 as ideal materials for further investigation of the underlying molecular mechanisms.

### 4.2. Role of Carbohydrate and Amino Acid Metabolism Pathways in Rye Cold Tolerance

Transcriptome sequencing has emerged as a powerful tool for elucidating the molecular mechanisms underlying plant cold tolerance. In our transcriptomic analysis of the “Winter” cultivar, 1643 differentially expressed genes (DEGs) were identified. Subsequent GO, KEGG, and GSEA enrichment analyses revealed significant enrichment of the carbohydrate metabolism (ko00630) and amino acid metabolism (ko00250) pathways under cold stress. These pathways exhibited substantial changes in the expression levels of key genes, suggesting that modulation of these metabolic routes is critical for the cold adaptation of rye.

Similar observations have been reported in other species. For example, eggplant under cold stress adjusts the expression of genes related to starch and sucrose metabolism, energy metabolism, redox reactions, and hormone signaling to maintain cellular function [26,27,28]. In cucumber seedlings, glucose pretreatment enhanced cold tolerance by modulating glycolysis and the tricarboxylic acid cycle [29]. Moreover, olive and Chinese cabbage maintain cellular osmotic balance under cold stress by regulating pathways involved in glucose, sucrose, and mannitol metabolism [30,31]. These studies collectively underscore the universal role of carbohydrate and amino acid metabolism in plant responses to low-temperature stress.

In rye, our results suggest that the pyruvate acid and dicarboxylic acid metabolism pathway (ko00630) plays a pivotal role in cold tolerance. Analysis of this pathway revealed 106 annotated genes, including upregulated candidates such as members of the *Serine hydroxymethyltransferase* (*SHM*) gene family and *acyl-activating enzyme (AAE)* genes, as well as downregulated genes like *Phosphoglycolate Phosphatase* (PGLP) and *Acetyl-CoA acetyltransferase (AACT)*. These expression changes likely alter the activities of key enzymes, thereby affecting the rate and direction of metabolic reactions and ultimately enhancing cold tolerance. Moreover, the alanine–aspartate–glutamate metabolism pathway displayed even more pronounced enrichment, with 7 of 10 candidate genes being upregulated. For instance, the *L-aspartate oxidase* (*NadB*) gene (*ScWN2R01G062200*) was upregulated approximately tenfold under cold stress, whereas the *Amidophosphoribosyltransferase* (*ASE*) gene (*ScWN3R01G472000*) was downregulated to about one-third of its original level. These alterations likely modulate the synthesis of key intermediates such as L-glutamine, L-glutamate, and mesaconate, which may in turn facilitate the flexible regulation of energy supply and osmotic balance under cold stress.

### 4.3. Application of Transcriptome Sequencing and Molecular Markers in Rye Cold Tolerance Research

Molecular marker technology plays a pivotal role in plant breeding by accelerating the selection process for stress tolerance traits. Transcriptome-based development of molecular markers offers distinct advantages, including direct reflection of gene expression changes, high-throughput screening capability, high accuracy, and clear functional associations [17,18,19,20,21,24]. In this study, we utilized transcriptomic data from the “Winter” cultivar to develop various molecular markers, including EST-SSR, EST-SNP, and KASP markers, thereby providing novel tools for marker-assisted selection in cold-tolerant rye breeding.

Specifically, we identified 10,846 EST-SSR loci and developed 13 universal EST-SSR primer pairs for rye, alongside detecting 250,116 EST-SNPs. Through SNP screening and qPCR validation, we further developed 7 KASP markers, among which KASP-665 effectively distinguished winter rye from spring rye by differentiating a T:T to T:C transition, suggesting a potential association with cold tolerance. Although not all KASP markers directly correlate with cold tolerance, they have proven useful for analyzing population genetic structure, parental selection, and heterosis prediction [32,33,34,35,36,37,38,39]. Collectively, these markers provide robust technical support for the breeding of cold-tolerant rye varieties and demonstrate a successful application of transcriptome sequencing in molecular marker development. This work lays a solid foundation for future studies aimed at further uncovering the molecular mechanisms of cold tolerance in rye.

## 5. Conclusions

In this study, we demonstrated that the rye cultivar “Winter” exhibits high and stable antioxidant enzyme activity under prolonged cold stress. Transcriptome sequencing revealed that the primary metabolic pathways responsive to low-temperature stress are the pyruvate acid and dicarboxylic acid metabolism pathway and the alanine–aspartate–glutamate metabolism pathway. Additionally, transcriptome-based screening allowed for the identification of SSR and SNP loci, leading to the development of 13 universal EST-SSR markers and 7 KASP markers for rye. Together, these transcriptomic data and molecular markers enhance our understanding of the physiological and biochemical mechanisms underlying cold adaptation in rye and offer significant potential for improving rye yields in cold regions through marker-assisted breeding.

## Figures and Tables

**Table 1:** Number of “Winter” rye EST-SSRs.

Chromosome Location	Number
Chromosome1R	1491
Chromosome2R	1628
Chromosome3R	1398
Chromosome4R	1448
Chromosome5R	1757
Chromosome6R	1374
Chromosome7R	1404
Unknown Chromosome	346
Total	10,846

## Data Availability

The original contributions presented in this study are included in the article. Further inquiries can be directed to the corresponding author.

## Appendix A

### Appendix A.1

**Table A1:** Transcriptome data filtering statistics of “Winter” rye.

Sample	RawData	CleanData (%)	Adapter (%)	LowQuality (%)	PolyA (%)	N (%)
D-CK-1	48,707,146	48,582,636 (99.74%)	13,498 (0.03%)	111,006 (0.23%)	0 (0.00%)	6 (0.00%)
D-CK-2	47,871,738	47,739,978 (99.72%)	14,448 (0.03%)	117,306 (0.25%)	0 (0.00%)	6 (0.00%)
D-CK-3	53,268,824	53,127,680 (99.74%)	15,382 (0.03%)	125,762 (0.24%)	0 (0.00%)	0 (0.00%)
D-12-1	58,454,228	58,295,122 (99.73%)	17,392 (0.03%)	141,712 (0.24%)	0 (0.00%)	2 (0.00%)
D-12-2	62,533,210	62,354,096 (99.71%)	19,844 (0.03%)	159,258 (0.25%)	0 (0.00%)	12 (0.00%)
D-12-3	58,815,098	58,660,926 (99.74%)	19,262 (0.03%)	134,906 (0.23%)	0 (0.00%)	4 (0.00%)

D-CK represents the winter control group, and D-12 represents the winter experimental group treated at 4 °C for 12 h, with three biological replicates per group.

### Appendix A.2

**Table A2:** Two important expression pathways in “Winter” rye.

Pathway ID	Pathway	KEGG_A Class	KEGG_B Class	Candidate Genes	Upgrade Candidate Genes	Down Candidate Genes	All Genes
ko00630	Glyoxylate and dicarboxylate metabolism	Metabolism	Carbohydrate metabolism	13	7	6	112
ko00250	Alanine, aspartate and glutamate metabolism	Metabolism	Amino acid metabolism	10	4	6	79

### Appendix A.3

**Table A3:** Analysis of 151 pairs of EST-SSR primers.

Categories	Numbers
Non-amplified band	8
Amplified band	143
Polymorphic band	93
Unitary strip	50

### Appendix A.4

**Table A4:** Primer information of “Winter” rye EST-SSR primers.

ID	SSR	Size	FORWARDPRIMER (5′-3′)	REVERSEPRIMER (5′-3′)	Tm (°C)	PRODUCT Size (bp)
Sc1R14	(CGCCGA)4	24	CCTCCTCCTCCTCCTCCTC	CCGAAGCTGTCGTAGTGGAT	57.1	253
Sc1R20	(CT)7	14	TCCTCCCCCTCTCTTCTCTC	ACATAACGGTCCGCAAACTC	55.4	212
Sc3R06	(TTCT)6	24	AGCTTCTGCTGGTCTGTGGT	AAGAATGCCAGAGCAGGCTA	56.3	184
Sc3R09	(CCGC)5	20	CAGCAGAGTCCAACAAACCC	AGCTGGGCGAGCTCTTGT	56.7	205
Sc4R02	(CCT)5	15	AACCTCGCTCCACCTCATCT	AATGGAAGGTGTCCGGGT	56.6	278
Sc4R08	(TA)6	12	GAGAGCCGTCACTTTTGGTC	TCACAGCAGAGATGAGGTCG	56.3	165
Sc4R18	(TG)8	16	AGCAAGCTGCTTCTCTGAGG	TTTGCTCACGACTTTGGTTG	53.4	244
Sc6R15	(CAG)7	21	ATCCAATGAATCAAGCCAGC	CATGGAGTGATGGTTAGGGG	53	278
Sc7R03	(GAA)7	21	GGGACTCTGACAACGACGAT	TAATGGTGGCCCAAGAAGAC	54.8	148
Sc1R02	(GAT)6	18	CTGGTGATACAGCCAACCCT	CGGATAGCACGGAGTACGTT	57	205
Sc1R03	(TTTC)5	20	AGGTTGTCATTTTCATGCCC	GAGCTGATCTTCAAGGCCAC	53.1	237
Sc4R04	(ACG)9	27	CTCCTCAAGTCCAGTGCTCC	TCCATCAGAGACAGCAGACG	56.8	233
Sc5R03	(GC)6	12	CAAATTTAGCCACCTCCCAA	CCTTTCTTTCCGCTTCCTCT	52.6	247

### Appendix A.5

**Table A5:** Analysis of 140 pairs of EST-SNP primers.

Categories	Numbers
Non-amplified band	56
Amplified band	84
Polymorphic band	54
Unitary strip	30

### Appendix A.6

**Table A6:** Primer information for 7 KASP molecular markers.

ID	Ref	Mut	FORWARD PRIMER1 (5′-3′)	REVERSE PRIMER1 (5′-3′)	Primer Common
KSAP-063	C	T	GCCGCGCCGGTGA	CGCCGCGCCGGTA	AGGAGAAGGTCGGGG AGGTGAA
KASP-050	C	A	CGCGGCTTGTGCAGCAGAC	CTCGCGGCTTGTGCAGCAGAA	GCAGCTTGGGGACATCGCCAAT
KASP-109	G	C	CGCCGGCGTGGCG	CGCCGGCGTGGCC	GATGTCGGCCTGGATCTTGCTGTA
KASP-126	T	C	CCACGCCATCGTCCTCGGT	CACGCCATCGTCCC GGC	CACTGGAGGAAGGTGCCGATGAA
KASP-424	A	G	GCCCCCAGCATCTTCGCCA	CCCCCAGCATCTTG CCG	TGATGAGCCACGACCCCCTGAA
KASP-396	C	T	CGGTGCACGCGCC GGC	CCGGTGCACGCGCC GGT	CCAAGCAGGCCCTTCTTCTCCTT
KASP-665	G	A	GCCGCCGGTGCG GGC	GGCCGCCGGTGCG GGT	AGGAGAAGCTGCCTGGCCACAA

### Appendix A.7

**Table A7:** Primer information of the wheat actin gene (TaActin) used as an internal reference.

Gene Name	Forward Primer	Reverse Primer
TaActin	ATGGCTGGAGAAGGATTC	CTCCTTAATGTCACGCACG

### Appendix A.8

**Table A8:** The experimental materials used for the developed molecular markers.

Number	The Experimental Materials
1	“Winter”
2	HZHM3
3	HZHM8
4	“Victory”
5	RUS1060 HZHM132
6	RUS1063 HZHM135
7	RUS1087 HZHM159
8	RUS1095 HZHM167
9	RUS1080 HZHM158
10	RUS110 HZHM72
11	RUS960 HZHM32
12	RUS1079 HZHM151
13	RUS1081 HZHM153
14	RUS169
15	RUS1082
16	RUS1064
17	RUS1091
18	RUS966
19	RUS1071
20	RUS1098
21	RUS1075
22	RUS1090
23	RUS964
24	RUS920
25	RUS1057
26	RUS1073
27	RUS1070
28	RUS1092
29	RUS961
30	RUS990
31	RUS1088
